# Is antibacterial treatment intensity lower in elderly patients? A retrospective cohort study in a German surgical intensive care unit

**DOI:** 10.1186/s12913-019-4204-0

**Published:** 2019-06-10

**Authors:** Dominik Beier, Christel Weiß, Michael Hagmann, Ümniye Balaban, Manfred Thiel, Verena Schneider-Lindner

**Affiliations:** 1 Department of Anesthesiology and Surgical Intensive Care Medicine, Medical Faculty Mannheim, Heidelberg University, Theodor-Kutzer-Ufer 1-3, 68167 Mannheim, Germany; 2 Department of Biometry and Statistics, Medical Faculty Mannheim, Heidelberg University, Theodor-Kutzer-Ufer 1-3, 68167 Mannheim, Germany; 3 Department of Community Health Sciences, University of Manitoba, S113 - 750 Bannatyne Avenue, Winnipeg, Manitoba R3E 0W3 Canada

**Keywords:** Antibacterials, ICU, Older patients, Electronic patient record, Zero-inflated Poisson regression

## Abstract

**Background:**

Demographic change concurrent with medical progress leads to an increasing number of elderly patients in intensive care units (ICUs). Antibacterial treatment is an important, often life-saving, aspect of intensive care but burdened by the associated antimicrobial resistance risk. Elderly patients are simultaneously at greater risk of infections and may be more restrictively treated because, generally, treatment intensity declines with age. We therefore described utilization of antibacterials in ICU patients older and younger than 80 years and examined differences in the intensity of antibacterial therapy between both groups.

**Methods:**

We analysed 17,464 valid admissions from the electronic patient data management system of our surgical ICU from April 2006 – October 2013. Antibacterial treatment rates were defined as days of treatment (exposed patient days) relative to patient days of ICU stay and calculated for old and young patients. Rates were compared in zero-inflated Poisson regression models adjusted for patients’ sex, mean SAPS II- and TISS-scores, and calendar years yielding adjusted rate ratios (aRRs). Rate ratios exceeding 1 represent higher rates in old patients reflecting greater treatment intensity in old compared to younger patients.

**Results:**

Observed antibacterial treatment rates were lower in patients 80 years and older compared to younger patients (30.97 and 39.73 exposed patient days per 100 patient days in the ICU, respectively). No difference in treatment intensity, however, was found from zero-inflated Poisson regression models permitting more adequate consideration of patient days with low treatment probability: for all antibacterials the adjusted rate ratio (aRR) was 1.02 (95%CI: 0.98–1.07). Treatment intensities were higher in elderly patients for penicillins (aRR 1.37 (95%CI: 1.26–1.48)), cephalosporins (aRR 1.20 (95%CI: 1.09–1.31)), carbapenems (aRR 1.35 (95%CI: 1.20–1.50)), fluoroquinolones (aRR 1.17 (95%CI: 1.05–1.30), and imidazoles (aRR 1.34 (95%CI: 1.23–1.46)).

**Conclusions:**

Elderly patients were generally less likely to be treated with antibacterials. This observation, however, did not persist in patients with comparable treatment probability. In these, antibacterial treatment intensity did not differ between younger and older ICU patients, for some antibacterial classes treatment intensity was even higher in the latter. Patient-level covariates are instrumental for a nuanced evaluation of age-effects in antibacterial treatment in the ICU.

**Electronic supplementary material:**

The online version of this article (10.1186/s12913-019-4204-0) contains supplementary material, which is available to authorized users.

## Background

Severe infectious complications such as sepsis are common and increasing in critically ill patients in intensive care units (ICUs) [1, 2]. Concurrently, the rising prevalence of multi-resistant organisms in ICUs forces clinicians to use antibacterial combinations and agents of last resort for management of such infections, thereby further fostering antimicrobial resistance development [3–5]. Antibacterial therapy thus places intensive care physicians in a worsening dilemma of providing adequate therapy for the individual while considering not only the good of the single patient but of the whole ICU [6]. This conflict especially applies to older patients who are on the one hand at greater risk for developing infections [7] and on the other hand may only experience limited benefit from aggressive therapy, especially at the end of life [6, 8]. Elderly patients may consequently receive a disproportionately large share of antibacterials and thus are a group of interest upon which to focus antibacterial utilization improvement activities, such as antibiotic stewardship programs. By contrast, general treatment intensity in ICUs declines with advancing age [9–13], although it is acknowledged that age alone should not be decisive for this [7, 9, 14]. These conflicting treatment tendencies make it unclear if current utilization of antibacterials in elderly ICU patients differs from that of younger ones. This question is of increasing importance given that the proportion of elderly patients requiring complex care is predicted to rise due to the demographic change [11, 15–18]. To the best of our knowledge, however, no studies have yet investigated differences in antibacterial therapy in older compared to younger ICU patients. Moreover, previous antibacterial drug utilization studies in ICUs have focused on total antibacterial consumption or impacts of antibiotic stewardship programs, while data quantifying antibacterial therapy based on individual patient data are scarce.

In this study we therefore compared antibacterial therapy in adult ICU patients above and below 80 years, a cut-off applied previously for studies in this setting [8–10, 13, 15, 16, 18]. We hypothesized that in our ICU elderly compared to younger patients more often require antibacterial treatment. In addition to all antibacterials we also separately assessed different antibacterial drug classes to detect potential differences in local therapy patterns between the two age groups, and to reveal differential utilization of antibacterials in our ICU associated with high resistance risk. Because in our ICU both patients with urgent and no need for antibacterial treatment are treated concomitantly, we accounted for this situation with adequate analytical methods.

## Methods

### Data source and cohort definition

The data used for this study were extracted from the patient data management system (IntelliVue Clinical Information Portfolio, Philips, Eindhoven, Netherlands) of the Department of Anesthesiology and Surgical Intensive Care Medicine at University Hospital Mannheim, Germany. The same data source has been used previously [19]. The available data consisted of anonymized electronic medical records on patients treated in our 26-bed surgical ICU from April 2006 to October 2013. Consistent with our aim of including the whole ICU-population during the study period in our analyses, the cohort was defined as all valid admissions of patients aged 18 years or older with known sex and age and a minimum stay of 10 min. Follow-up started on ICU admission or the beginning of electronic records, whichever was earlier, and continued until the earliest of discharge, death in the ICU, or end of available data. Though patients could be admitted more than once to the ICU during the study period, we refer to individual admissions as patients. Age was categorized into < 80 and ≥ 80 years.

The referring department of each admission was extracted. We additionally analysed microbiological test results of blood cultures and bronchoalveolar lavages from all patients in the ICU between 26/03/2007 and 31/12/2013 to establish time trends in bacteria isolated on our ward. In excluding other samples, such as routine admission swabs, we focused on results with relevance for antibacterial treatment. Furthermore, the Charlson comorbidity score [20] was determined from ICD-10 codes from administrative hospital discharge data [21] for all patients with indicator for treatment in the ICU during the study period. However, as there was no individual linkage to the electronic medical records of the ICU, this information served for comparative description only and could not be included in the analyses on antibacterial treatment.

### Antibacterial exposure and treatment measures

Entries of antibacterials in the electronic medical record during follow-up were identified and categorized into antibacterial drug classes according to the WHO Anatomical Therapeutic Chemical Classification System (ATC). The following classes were administered: tetracyclines (J01AA12), penicillins (J01CA01, J01CA04, J01CA10, J01CA12, J01CF04, J01CF05), cephalosporins (J01DB04, J01 DC02, J01DD01, J01DD02, J01DD04, J01DE01), carbapenems (J01DH02, J01DH03, J01DH04, J01DH51), macrolides (J01FA01, J01FA09), lincosamides (J01FF01), aminoglycosides (J01GB01, J01GB03, J01GB06), fluoroquinolones (J01MA01, J01MA02, J01MA12, J01MA14), glycopeptides (J01XA01), imidazoles (J01XD01), and oxazolidinones (J01XX08). In addition, some other antibacterials were administered which do not belong to one of the aforementioned classes (colistin (J01XB01), fosfomycin (J01XX01), daptomycin (J01XX09), and rifampicin (J04AB02)). The 368 records for these were only considered in analyses of all antibacterials.

There are several possibilities to measure antibacterial exposure and consumption, all of which have their advantages and disadvantages [22–24]. In addition to defining patients with at least one record of any antibacterial during their ICU stay as exposed patients, we used three antibacterial utilization measures (Additional file 1: Figure S1): (1) *Antibacterial prescriptions* represented individual entries of antibacterials in our electronic patient record. These comprised separate doses, e.g., infusions or flow rates of syringe pumps. A patient could therefore have multiple entries of the same antibacterial on a given day depending on how the daily dose was split up. Consequently, ranking and comparability of prescriptions between classes is limited. We therefore also analysed (2) *antibacterial therapy days*, defined as days in which antibacterial prescriptions of one or more particular classes were recorded. If a patient received antibacterials of two or more different drug classes in one calendar day, two or more therapy days were counted. Thus, this measure reflects both combination and change of antibacterial classes and mainly served for illustration of time trends in treatment complexity. Finally, we defined (3) *exposed patient days* as calendar days in which at least one antibacterial agent, irrespective of the class, was administered, i.e., at least one antibacterial prescription was recorded. This measure, relative to days of patient stay, allowed calculation of antibacterial exposure rates. A calendar day in which a patient stayed at least 1 min in the ICU was defined as a *patient day*. Consequently, a patient could have more antibacterial prescriptions than therapy days and more therapy days than exposed patient days.

These measures were determined for all patients and all antibacterials. In addition, all antibacterials excluding the macrolide erythromycin due to its nearly exclusive off-label use as a prokinetic agent in our ICU were quantified accordingly. Furthermore, exposed patient days were counted for all antibacterial drug classes separately. Macrolides were analysed both with and without erythromycin, with clarithromycin being the only other macrolide administered to our cohort. In the category ‘all antibiotics’ and ‘all antibiotics except erythromycin’ the abovementioned other antibacterials not belonging to one of the drug classes were also included.

### Cofactors

The respective age group was the main covariate of interest. In addition, we included the patient’s sex, the year of treatment, severity of the acute illness as expressed by the Simplified Acute Physiology Score II (SAPS II) [25], as well as the Therapeutic Intervention Scoring System (TISS) [26] assessing intensity of patient care as adjusting factors in the analysis. As usual in German ICUs, a simplified TISS-score is recorded in our electronic patient record which contains the ten most intensive therapeutic measures (‘TISS-10’). SAPS II-scores were re-calculated by subtracting the points contributed to the overall SAPS II-score by the age category to allow comparability of actual severity of acute illness between the age groups [10, 12, 13, 15]. For every patient the means of available daily SAPS II- and TISS-10-scores of a given admission were calculated. Missing scores were set to 0, i.e., for SAPS II parameters normal values and for TISS-10 no interventions were assumed.

### Strategies for addressing potential confounding

The statistical models were adjusted for the above-mentioned cofactors to minimize differences in disease severity or other characteristics impacting infection risk between the age groups that are potentially associated with antibacterial treatment (potential confounding). These cofactors mainly reflected acute disease severity and intensity of therapeutic measures received by the patient. The SAPS II-score, however, additionally considered metastatic malignant tumours, haematological malignancy and AIDS and thus also served as a rough surrogate for significant chronic comorbidity. Our models did not include the treatment indication, i.e., the type of infection and causative bacterial agent. They were not adjusted for individual chronic comorbid conditions affecting infection risk, such as diabetes, and immunocompromised states, e.g., due to drug therapy. The models also contained no information on body weight, smoking, or alcohol intake.

### Statistical analysis

We examined differences between medians (due to skewed distributions) and frequencies for basic cohort parameters for all patients and both age groups with Mann-Whitney-U-tests and Pearson’s Chi^2^-tests respectively. We used standard Poisson regression to calculate the rates of exposed patient days (numerator of rate) per 100 patient days (denominator of rate) with 95% confidence intervals (95%CI) for all patients and for each age group separately. Furthermore, we calculated rate ratios for patients aged ≥80 compared to < 80 years of age yielding exposed patient days for patients ≥80 per 100 exposed patient days for patients < 80 by using zero-inflated Poisson regression. We chose the zero-inflation model since most patients of our cohort did not receive any antibacterial treatment and so had zero exposed patient days, i.e., there were excess zeros in the data.

The rates and rate ratios comprise the whole study period and were calculated for all antibacterial drug classes, all drug classes except erythromycin and for each drug class separately. The rate ratios were both unadjusted and adjusted for the patients’ sex, mean SAPS II- and TISS-score, and year of treatment. As a sensitivity analysis the subgroup of patients with more than 48 h of ICU treatment was described and analysed in the same way, with erythromycin excluded from the exposure definition. The analyses were conducted with SAS 9.4 (SAS Institute, Inc., Cary, NC).

## Results

Of 17,464 valid admissions to our ICU between April 2006 and October 2013 the majority were male (57.1%) and 2640 (15.1%) belonged to the age group of 80 and above (Table 1). Mean age was 63.8 ± 16.3 years and the median length of stay 0.9 days (interquartile range (IQR): 0.8–2.6), with a higher median of 2.8 (IQR: 0.8–8.3) in the 1448 (8.3%) non-survivors. Slightly more than one third had at least one valid SAPS II- and TISS-10-score (36.3%). The median of the means was 20.1 (IQR: 13.3–28.9) for SAPS II without age-points and 9.7 (IQR: 5.0–12.5) for TISS-10. Patients with a SAPS II- and TISS-score contributed to 72.2% of total patient days. Most admissions of the cohort and in young patients were neurosurgical, whereas older patients were most often transferred from orthopaedics (Additional file 1: Table S1). For all ICU patients the median Charlson score was 2, (IQR: 0–3) with higher scores in old compared to young patients (median 2, IQR: 1–4 vs. median 2, IQR: 0–3, *p* < 0.0001).

**Table 1:** Basic characteristics of the ICU cohort and patients treated with antibacterials

Characteristic	All patients	< 80 years	≥80 years	*p*-value^*^
Full cohort				
Number of patients	17,464	14,824 (84.9%)	2640 (15.1%)	
Patient days	76,424	67,480	8944	
Length of stay (median, IQR (days))	0.9 (0.8–2.6)	0.9 (0.8–2.7)	0.9 (0.8–2.0)	0.0004
Female patients	7498 (42.9%)	5875 (39.6%)	1623 (61.5%)	< 0.0001
ICU mortality rate	1448 (8.3%)	1136 (7.7%)	312 (11.8%)	< 0.0001
SAPS II (median of means, IQR)^a^	20.1 (13.3–28.9)	19.9 (13.0–28.6)	22.0 (15.0–30.0)	< 0.0001
TISS-10 (median of means, IQR)	9.7 (5.0–12.5)	10.0 (5.7–12.6)	7.5 (5.0–11.1)	< 0.0001
Patients treated with antibacterials				
Number of patients	5785 (33.1%^b^)	5060 (34.1%^c^)	725 (27.5%^c^)	< 0.0001
Patient days	44,936	40,831	4105	
Length of stay (median, IQR (days))	2.2 (0.9–8.7)	2.3 (0.9–9.1)	2.0 (0.9–5.7)	0.0142
Female patients	2047 (35.4%)	1689 (33.4%)	358 (49.4%)	< 0.0001
ICU mortality rate	869 (15.0%)	726 (14.4%)	143 (19.7%)	0.0002
SAPS II (median of means, IQR)^a^	24.6 (17.5–32.7)	24.4 (17.3–33.0)	25.8 (18.5–31.5)	0.4291
TISS-10 (median of means, IQR)	10.7 (7.5–14.1)	10.8 (7.8–14.4)	9.0 (5.0–12.1)	< 0.0001
Exposed patient days	29,578	26,808	2770	
Number of Antibacterial prescriptions	190,689	172,963	17,726	
Therapy days	52,342	47,630	4712	

^*^from Chi^2^-Test for proportions and Mann-Whitney-U-Test for continuous variables (two-sided with significance level of *p* < 0.05)

^a^SAPS II calculated without age points

^b^Percent of full cohort

^c^Percent of age group

About one third of patients were treated with antibacterials during their ICU stay (Table 1). Of these, an even larger proportion than in the full cohort was male (64.6%) and younger than 80 years (87.5%). Mean age was thus slightly lower compared to the whole cohort (62.4 ± 16.2 years), whereas length of stay (median 2.2 days (IQR: 0.9–8.7) was greater. While in the full cohort the proportion of females in patients aged 80 or above was larger than that of males, only about half of the elderly patients treated with antibacterials were female.

More than 190,000 antibacterial prescriptions were recorded for all patients during the study period (ca. 140,000 when excluding erythromycin), for which penicillins accounted for the most (Table 2). This is likely due to predominant administration of the penicillin class member piperacillin with syringe pumps, for which frequent records are created. In contrast, the largest number of exposed patients received cephalosporins, while most exposed patient days were observed for macrolides (predominantly erythromycin).

**Table 2:** Antibacterial treatment measures for both age groups

Drug class	Patients < 80 years			Patients ≥ 80 years		
	Exposed patients	Prescriptions	Exposed patient days	Exposed patients	Prescriptions	Exposed patient days
All antibacterials	5060 (100%^a^)	172,963 (100%^b^)	26,808 (100%^a^)	725 (100%^a^)	17,726 (100%^b^)	2770 (100%^a^)
All antibacterials except erythromycin	4673 (92.4%)	125,855 (72.8%)	22,597 (84.3%)	680 (93.8%)	14,567 (82.2%)	2477 (89.4%)
Tetracyclines	93 (1.8%)	1098 (0.6%)	631 (2.4%)	6 (0.8%)	67 (0.4%)	37 (1.3%)
Penicillins	1349 (26.7%)	63,655 (36.8%)	5640 (21.0%)	211 (29.1%)	8741 (49.3%)	806 (29.1%)
Cephalosporines	1711 (33.8%)	10,901 (6.3%)	6208 (23.2%)	248 (34.2%)	1041 (5.9%)	689 (24.9%)
Carbapenems	890 (17.6%)	14,973 (8.7%)	5787 (21.6%)	81 (11.2%)	961 (5.4%)	389 (14.0%)
Macrolides	1574 (31.1%)	47,311 (27.4%)	10,366 (38.7%)	168 (23.2%)	3183 (18.0%)	734 (26.5%)
Clarithromycin	34 (0.7%)	203 (0.1%)	124 (0.5%)	7 (1.0%)	24 (0.1%)	17 (0.6%)
Lincosamides	219 (4.3%)	966 (0.6%)	465 (1.7%)	40 (5.5%)	194 (1.1%)	95 (3.4%)
Aminoglycosides	85 (1.7%)	689 (0.4%)	361 (1.3%)	6 (0.8%)	26 (0.1%)	16 (0.6%)
Fluoroquinolones	1474 (29.1%)	13,954 (8.1%)	4950 (18.5%)	198 (27.3%)	1447 (8.2%)	556 (20.1%)
Glycopeptides	88 (1.7%)	473 (0.3%)	297 (1.1%)	9 (1.2%)	28 (0.2%)	17 (0.6%)
Imidazoles	1483 (29.3%)	12,601 (7.3%)	5297 (19.8%)	230 (31.7%)	1762 (9.9%)	768 (27.7%)
Oxazolidinones	493 (9.7%)	5771 (3.3%)	3163 (11.8%)	36 (5.0%)	251 (1.4%)	147 (5.3%)

^a^Sum of proportions of antibacterial classes exceeds 100% as patients could have been exposed to more than one class

^b^Sum of prescriptions is slightly lower than 100% as some rarely used antibacterials were not analysed as separate classes, see methods section for details

In patients 80 years and older the largest proportion was exposed to cephalosporins, imidazoles and penicillins, in the younger group the largest fraction of patients was exposed to cephalosporins, macrolides and imidazoles. Antibacterial prescriptions, however, were mostly recorded for penicillins and macrolides in both age groups. Penicillins, followed by imidazoles and macrolides, accounted for the most exposed patient days in elderly patients, whereas younger patients were by far most often exposed to macrolides, cephalosporins and carbapenems.

Overall, in the cohort there were 38.70 (95%CI: 38.26–39.15) exposed patient days per 100 patient days (global rate), and 32.81 (95%CI: 32.41–33.22) exposed patient days per 100 patient days when excluding erythromycin (Table 3). The rate for patients aged < 80 years was higher (39.73 (95%CI: 39.25–40.21)) than the rate for older patients (30.97 (95%CI: 29.84–32.15)). These observed rates with confidence intervals from an unadjusted standard Poisson model reflect the actual status quo of antibacterial exposure in our ICU for all patients and both age groups. In contrast, the rate ratios of the zero-inflated Poisson-model can be interpreted as the intensity of treatment in patients above 80 compared to those below 80 years for those patients who were likely to be treated. The exposure rate ratio (RR) adjusted for sex, mean SAPS II- and TISS-scores, and year of treatment for all antibacterials was 1.02 (95%CI: 0.98–1.07) indicating a comparable treatment intensity in both age groups. For all antibacterials except erythromycin (adjusted RR 1.07 (95%CI: 1.02–1.12) and all individual antibacterial drug classes except for macrolides, aminoglycosides and glycopeptides adjusted exposure rate ratios reflected significantly higher rates in older patients or a greater treatment intensity compared to patients younger than 80 years. An analysis restricted to patients with more than 48 h of stay in our ICU yielded highly robust results (Additional file 1: Tables S3 and S4). In this group, only 59 or 1.2% had neither SAPS II- nor TISS-scores.

**Table 3:** Rates and rate ratios with 95%CIs for antibacterial therapy by drug class

Drug class	Observed rate (whole ICU cohort) ^a^	Observed rate patients aged < 80 years^a^	Observed rate patients aged ≥ 80 years^a^	Unadjusted rate ratio ≥ 80 vs. < 80^b^	Adjusted rate ratio ≥ 80 vs. < 80^c^
All Antibacterials	38.70 (38.26–39.15)	39.73 (39.25–40.21)	30.97 (29.84–32.15)	0.99 (0.94–1.03)	1.02 (0.98–1.07)
All antibacterials except erythromycin	32.81 (32.41–33.22)	33.49 (33.05–33.93)	27.69 (26.63–28.81)	1.05 (1.00–1.10)	1.07 (1.02–1.12)
Tetracyclines	0.87 (0.81–0.94)	0.94 (0.86–1.01)	0.41 (0.30–0.57)	2.26 (1.59–3.22)	1.87 (1.25–2.81)
Penicillins	8.43 (8.23–8.64)	8.36 (8.14–8.58)	9.01 (8.41–9.66)	1.41 (1.29–1.53)	1.37 (1.26–1.48)
Cephalosporins	9.02 (8.81–9.24)	9.20 (8.97–9.43)	7.70 (7.15–8.30)	1.22 (1.11–1.34)	1.20 (1.09–1.31)
Carbapenems	8.08 (7.88–8.28)	8.58 (8.36–8.80)	4.32 (3.91–4.77)	1.29 (1.15–1.44)	1.35 (1.20–1.50)
Macrolides	14.52 (14.26–14.80)	15.36 (15.07–15.66)	8.21 (7.63–9.82)	0.96 (0.88–1.04)	0.97 (0.90–1.06)
Clarithromycin	0.18 (0.16–0.22)	0.18 (0.15–0.22)	0.19 (0.12–0.31)	1.17 (0.64–2.14)	1.02 (0.51–2.04)
Lincosamides	0.73 (0.67–0.80)	0.69 (0.63–0.75)	1.06 (0.87–1.30)	1.41 (1.08–1.83)	1.40 (1.05–1.86)
Aminoglycosides	0.49 (0.45–0.55)	0.53 (0.48–0.59)	0.18 (0.11–0.29)	1.12 (0.61–2.04)	1.11 (0.61–2.02)
Fluoroquinolones	7.20 (7.02–7.40)	7.34 (7.13–7.54)	6.22 (5.72–6.76)	1.24 (1.12–1.37)	1.17 (1.05–1.30)
Glycopeptides	0.41 (0.37–0.46)	0.44 (0.39–0.49)	0.19 (0.12–0.31)	2.16 (1.14–4.10)	1.69 (0.90–3.20)
Imidazoles	7.94 (7.74–8.14)	7.85 (7.64–8.06)	8.59 (8.00–9.22)	1.36 (1.25–1.48)	1.34 (1.23–1.46)
Oxazolidinones	4.33 (4.19–4.48)	4.69 (4.53–4.85)	1.64 (1.40–1.93)	1.22 (1.02–1.47)	1.30 (1.08–1.56)

^a^Exposed patient days per 100 patient days

^b^Zero-inflated Poisson-regression

^c^Zero-inflated Poisson-regression adjusted for mean SAPS II- and TISS-scores, sex, and year of treatment

For all antibacterials there was no overall time trend in treatment intensity and no difference in comparative treatment intensity for the age groups over time as both the year and interaction of year and age did not reach statistical significance in the zero-inflated Poisson model. This is congruent with positive microbiological test results from blood cultures and bronchioalveolar lavage. Their number increased steadily during the study period, but the most common infectious agents were consistently identified as Staphylococci, *E. coli*, and *Candida albicans* (Additional file 1: Table S2). Treatment intensity was slightly lower, however, for female compared to male patients (adjusted RR 0.96 (95%CI: 0.93–0.98)).

**Fig. 1:**
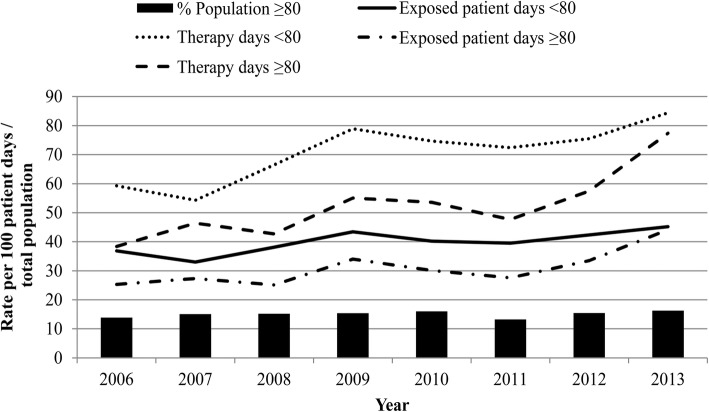
Time trends of exposed patient days and treatment days by age group, and percentage of elderly patients in the ICU cohort. Proportion of patients aged ≥80 years from a German 26-bed surgical ICU based on 17,464 admissions from April 2006 to October 2013. Antibacterial treatment trends are reflected by the fraction of therapy days (sum of different classes of which antibacterial agents were administered in a day) per 100 patient days and exposed patient days (days of ICU stay in which at least one antibacterial agent irrespective of the class was administered) per 100 patient days

While the proportion of patients aged ≥80 years remained stable during the study period at around 15% in our ICU (Fig. 1), in both age groups the number of exposed patient days increased relative to 100 patient days. There was an even greater increase in antibacterial treatment days relative to patient stay, reflecting increasing complexity of treatment. Except for the years 2007 and 2008 the same trends were observed for both age groups, although the rates for patients < 80 years were consistently higher.

## Discussion

During the study period about one in three admissions to our ICU received antibacterials and one in eight was 80 years or older. Antibacterial prescriptions, antibacterial therapy days and exposure rate to antibacterials were all lower in older compared to younger patients. However, alongside high-risk patients our ICU admits patients for routine post-surgical surveillance with low infection risk who are predominantly elderly and contribute numerous person days free of antibacterial exposure. We therefore accounted for the baseline probability of antibacterial treatment with zero inflated Poisson regression. The resulting adjusted exposure rate ratios indicated no difference for all antibacterials, i.e., global treatment intensity was comparable, for most antibacterial classes there was even evidence for higher treatment intensity in elderly patients.

Based on exposure rates, the most often used classes in our ICU during the study period were macrolides, cephalosporins, penicillins and carbapenems. Ignoring macrolides - predominantly erythromycin used off-label as prokinetic agent - our findings are consistent with other studies reporting β-lactams as most often used, with penicillins in a French [27] and a German ICU [28], and highest consumption of cephalosporins followed by penicillins, carbapenems, and fluoroquinolones in Swedish ICUs, [29]. In a Spanish multi-centre study also β-lactams as a whole, carbapenems, and fluoroquinolones were most widely used [30].

In contrast to our findings, previous studies on mechanical ventilation have revealed less aggressive therapy and less ICU admissions for the elderly even after adjustment for disease severity [9, 10, 12, 13].

The discrepancy between observed and adjusted comparative antibacterial exposure can be reconciled as both results representing different aspects of the antibacterial treatment process, with the former reflecting treatment decision and the latter treatment implementation. Our results thus suggest there is no differential treatment of elderly patients once the treatment decision is made. The decision for treatment initiation, however, appears to be dependent on age or factors strongly associated with age. This includes the association of age with both a low treatment need, particularly in patients admitted for short-term routine post-surgical recovery, as well as with a decision against treatment. The greater difference between observed exposure rates in old and young patients regardless of treatment duration compared to rates from those with > 48 h of treatment supports that the first scenario was probably true for a significant proportion of old patients. Unfortunately, information on life-sustaining treatment limitations was not at our disposal to distinguish both scenarios definitively. We expect that younger and older patients with a priori explicit strong wish not to be aggressively treated are unlikely to be admitted to our surgical ICU. However, information on treatment preferences is often not sought before ICU admission [31], but, as varying by centre [32], may concern a significant proportion of elderly [33], potentially leading to a lower treatment probability for elderly in our study. Indeed, given their higher mortality in our ICU, for elderly probably more often the question arose whether life-prolonging therapy was still useful and in the patient’s interest [6, 8].

The lower treatment intensity in female patients may reflect sex-specific differences in infectious disease incidence but also differences in treatment implementation due to other causes. While some studies report contradictory results on differences in general treatment intensity in ICUs [34–37], Nachtigall et al. [37] did not find sex-based differences in antibacterial therapy in a sepsis cohort.

To our knowledge no previous study has explicitly focused on age-dependent differences in antibacterial therapy in ICU patients. Instead of aggregated data often used for antibacterial drug utilization studies we analysed patient-level data. We could thereby account for potential confounding in the age-group comparison by considering individual disease severity using the SAPS II-score together with therapeutic intensity reflected by TISS-10. Our findings of an at least comparable antibacterial treatment intensity in older ICU patients were confirmed in analyses restricted to patients with > 48 h of ICU treatment, who can be considered as high-risk for infection. This supports use of the zero-inflated Poisson model for all patients which facilitates a comparison to ICU-wide descriptive antibacterial utilization data. Moreover, this suggests general utility of this method for situations when a two-stage treatment process is likely but restriction of analyses to a subgroup with high treatment probability is not desirable or possible.

Our results provide a snapshot of antibacterial utilization in the ICU setting, treatment indication, however, was unknown. A distinction between ICU-imported and ICU-acquired infections was also not possible and the fraction of empirical antibacterial treatments is unknown. However, as the vast majority (83.3%) of patients treated for > 48 h received antibacterials within their first 48 h, a significant proportion of infections present on admission or soon thereafter is likely. Moreover, as our results reflect a single surgical ICU in Germany their generalizability is limited. Infectious disease incidence and infectious agents expectedly vary in other intensive care settings, such as neurological or cardiac surgery ICUs, and the difference between age groups may vary accordingly. In addition, antibacterial prescription practice can already differ between ICUs of the same country [24, 29]. Conclusions beyond German ICUs should be drawn even more cautiously since health care systems and services vary substantially across Western Europe and North America [38]. This variation also affects the ICU admission process [39]. Triage decisions lead to selective admission of elderly expected to benefit from admission who are likely more often treated than elderly refused ICU admission. It is unknown if our local triage was more or less restrictive than elsewhere and information on refused patients was not available. Thus, interpretation of our results requires consideration of the fact that they are only based on patients admitted according to local decision practice. Nevertheless, in addition to the cohort characteristics, the information on referring departments and microbiological isolates from our ICU provides some context for external comparisons.

While a binary age classification facilitated a simple interpretation of the exposure rate ratios, in further studies a greater number of age subgroups should be investigated, e.g., as previously defined as young-old (65–74 years), old-old (75–84 years), and very-old (> 84 years) [40]. Moreover, future investigations should include information on potential confounders such as antibacterial treatment indication and individual comorbidity, particularly diabetes and malignant diseases, immunosuppressant medication, as well as body weight, smoking and alcohol intake. A possible time-dependency of the treatment difference in the age groups based on length of stay should be considered. Future studies should also distinguish ICU-imported and ICU-acquired infections.

## Conclusion

In our surgical ICU elderly patients were less often treated with antibacterials than patients younger than 80 years. In those likely to be treated, however, intensity was at least similar in older compared to younger patients. These findings support differentiation between age as a factor for treatment decision and treatment implementation. This requires confirmation in studies from other surgical and non-surgical ICUs, which ideally should include data on treatment indication.

## Additional file

Additional file 1: **Figure S1.** Illustration of antibacterial utilization measures. **Table S1.** Referring departments of the ICU cohort. **Table S2.** Most common microbiological isolates from blood cultures and bronchioalveolar lavages 2006–2013. **Table S3.** Basic characteristics of ICU patients with length of stay > 48 h, all and those treated with antibacterials excluding erythromycin. **Table S4.** Rate ratios for antibacterial classes (patients with length of stay > 48 h). (DOC 135 kb)

## Data Availability

The data that support the findings of this study consist of individual patient records from our ICU which are subject to strict German data protection laws and regulations and so are not publicly available. We have been granted permission to analyse these data, after anonymization, on the hospital premises from the data protection officer of the University Medical Center Mannheim.

## Abbreviations

aRR: adjusted rate ratio
ATC: Anatomical Therapeutic Chemical (Classification System)
CI: Confidence interval
ICU: Intensive care unit
IQR: Interquartile range
RR: Rate ratio
SAPS II: Simplified Acute Physiology Score II
TISS: Therapeutic Intensity Scoring System
WHO: World Health Organization
