# Supplementary material for: Is antibacterial treatment intensity lower in elderly patients? A retrospective cohort study in a German surgical intensive care unit
Source: BMC Health Serv Res. 2019 Jun 10;19:367. doi: 10.1186/s12913-019-4204-0 (PMC6558678; doi:10.1186/s12913-019-4204-0)
Supplement: Supplementary file 1 — Figure S1. Illustration of antibacterial utilization measures. Table S1. Referring departments of the ICU cohort. Table S2. Most common microbiological isolates from blood cultures and bronchioalveolar lavages 2006–2013. Table S3. Basic characteristics of ICU patients with length of stay > 48 h, all and those treated with antibacterials excluding erythromycin. Table S4. Rate ratios for antibacterial classes (patients with length of stay > 48 h). (DOC 135 kb) [file 12913_2019_4204_MOESM1_ESM.doc]

**Is antibacterial treatment intensity lower in elderly patients? A retrospective cohort study in a German surgical intensive care unit.**

Dominik Beier, Christel Weiß, Michael Hagmann, Ümniye Balaban, Manfred Thiel, Verena Schneider-Lindner

**Additional file 1**

This section contains an illustration of the antibacterial utilization measures described in the methods section (Additional file 1: Figure S1) and provides additional contextual information on the surgical ICU (Additional file 1: Tables S1 and S2) and results of analyses restricted to patients with a minimum duration of 48 hours of ICU treatment (Additional file 1: Tables S3 and S4).


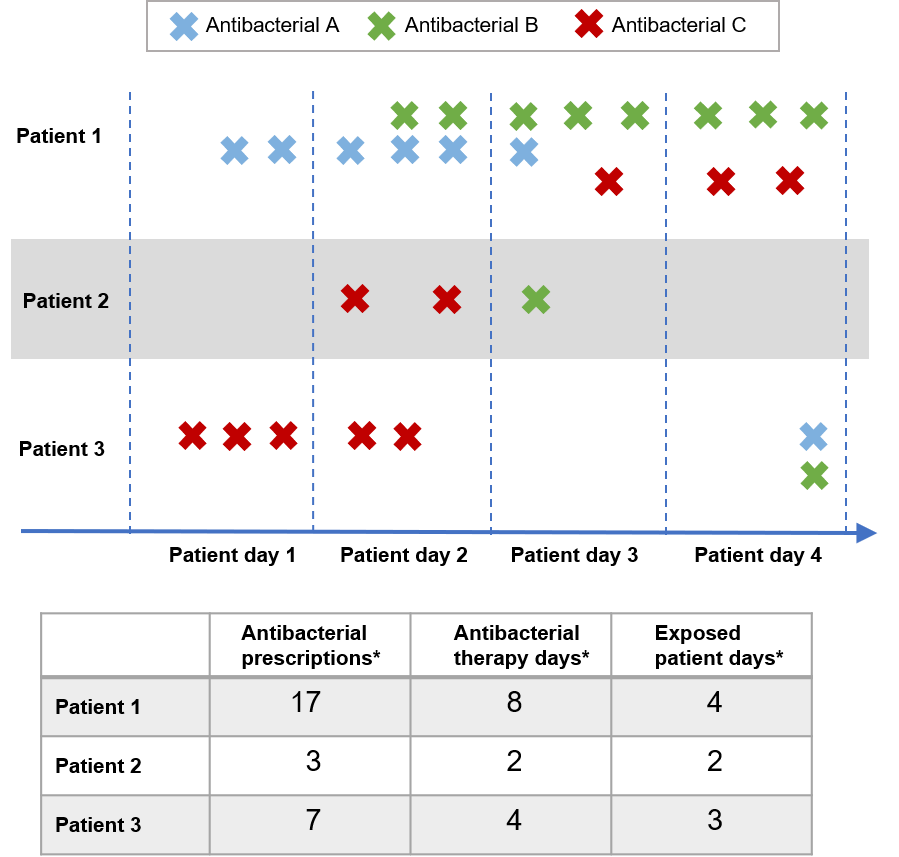


*per 4 patient days

**Additional file 1: Figure S1: Illustration of antibacterial utilization measures.**

Exemplary treatment scenarios of 3 fictitious patients for 4 days of ICU treatment and 3 different antibacterials. The corresponding utilization measures for the 4 patient days for each of the 3 fictitious patients are provided in the table below the diagram. The definitions of the utilization measures are described in the methods section of the main manuscript.

**Additional file 1: Table S**1: Referring departments of the ICU cohort

| **Department** | **All admissions** | **Admissions  <80 years** | **Admissions  ≥80 years** |
| --- | --- | --- | --- |
| All | 17,464 (100%) | 14,824 (100%) | 2640 (100%) |
| Neurosurgery | 4088 (23.4%) | 3677 (24.8%) | 411 (15.6%) |
| Orthopaedics | 2517 (14.4%) | 1845 (12.5%) | 672 (25.5%) |
| Urology | 2005 (11.5%) | 1811 (12.2%) | 194 (7.4%) |
| General surgery | 1816 (10.4%) | 1531 (10.3%) | 285 (10.8%) |
| Traumatology | 1469 (8.4%) | 1152 (7.8%) | 317 (12.0%) |
| Others* | 1559 (8.9%) | 1415 (9.6%) | 144 (5.5%) |
| More than one department | 834 (4.8%) | 737 (5.0%) | 97 (3.7%) |
| Unknown | 3176 (18.2%)^**^ | 2656 (17.9%) | 520 (19.7%) |

* Including ENT, gynaecology and non-surgical departments like internal medicine

**Of these 2571 (81.0%) had a length of stay of ≤ 48h

**Additional file 1: Table S2: Most common microbiological isolates from blood cultures and bronchioalveolar lavages 2006-2013**

| **Infectious agent** | **2006** | **2007** | **2008** | **2009** | **2010** | **2011** | **2012** | **2013** | **2006-2013** |
| --- | --- | --- | --- | --- | --- | --- | --- | --- | --- |
| Total number of isolates | 98 (100%) | 39 (100%) | 61 (100%) | 235 (100%) | 399 (100%) | 502 (100%) | 590 (100%) | 642 (100%) | 2566 (100%) |
| Staphylococcus epidermidis | 0 (0.0%) | 2 (5.1%) | 0 (0.0%) | 4 (1.7%) | 34 (8.5%) | 84 (16.7%) | 100 (17.0%) | 123 (19.2%) | 347 (13.5%) |
| Staphylococcus aureus | 26 (26.5%) | 6 (15.4%) | 18 (29.5%) | 36 (15.3%) | 65 (16.3%) | 39 (7.8%) | 66 (11.2%) | 59 (9.2%) | 315 (12.0%) |
| Staphylococcus, CONS* | 18 (18.4%) | 16 (41.0%) | 24 (39.3%) | 59 (25.1%) | 69 (17.3%) | 2 (0.4%) | 7 (1.2%) | 11 (1.7%) | 206 (7.3%) |
| Escherichia coli | 10 (10.2%) | 4 (10.3%) | 4 (6.6%) | 26 (11.1%) | 36 (9.0%) | 31 (6.2%) | 36 (6.1%) | 56 (8.7%) | 203 (7.8%) |
| Candida albicans | 6 (6.1%) | 1 (2.6%) | 0 (0.0%) | 16 (6.8%) | 38 (9.5%) | 50 (10.0%) | 52 (8.8%) | 38 (5.9%) | 201 (7.8%) |
| Enterobacter cloacae | 6 (6.1%) | 1 (2.6%) | 1 (1.6%) | 7 (3.0%) | 10 (2.5%) | 18 (3.6%) | 14 (2.4%) | 19 (3.0%) | 76 (3.0%) |
| Candida glabrata | 4 (4.1%) | 0 (0.0%) | 5 (8.2%) | 8 (3.4%) | 16 (4.0%) | 22 (4.4%) | 12 (2.0%) | 9 (1.4%) | 76 (3.0%) |
| Klebsiella pneumoniae | 1 (1.0%) | 1 (2.6%) | 0 (0.0%) | 4 (1.7%) | 7 (1.8%) | 18 (3.6%) | 21 (3.6%) | 20 (3.1%) | 72 (2.8%) |
| Enterococcus faecalis | 0 (0.0%) | 0 (0.0%) | 2 (3.3%) | 6 (2.6%) | 10 (2.5%) | 12 (2.4%) | 19 (3.2%) | 22 (3.4%) | 71 (2.8%) |
| Enterococcus faecium | 0 (0.0%) | 0 (0.0%) | 0 (0.0%) | 6 (2.6%) | 4 (1.0%) | 14 (2.8%) | 20 (3.4%) | 26 (4.1%) | 70 (2.7%) |
| Others | 28 (28.6%) | 8 (20.5%) | 7 (11.5) | 63 (26.8%) | 110 (27.6%) | 212 (42.2%) | 243 (41.2%) | 259 (40.3%) | 956 (37.3%) |

* coagulase-negative staphylococci

**Additional file 1: Table S3: Basic characteristics of ICU patients with length of stay > 48 hours, all and those treated with antibacterials excluding erythromycin**

| **All >48 hours** | | | | |
| --- | --- | --- | --- | --- |
| **Characteristic** | **All patients** | **<80 years** | **≥80 years** | **p-value^*^** |
| Number of patients | 4892 | 4225 (86.4%) | 667 (13.6%) |  |
| Patient days | 51,523 | 46,740 | 5053 |  |
| Length of stay (median, IQR (days)) | 6.0 (3.4 – 11.5) | 6.5 (3.5-12.1) | 4.6 (2.9-7.8) | <0.0001 |
| Female patients | 2006 (41.0%) | 1633 (38.7%) | 373 (55.9%) | <0.0001 |
| ICU mortality rate | 830 (17.0%) | 683 (16.2%) | 147 (22.0%) | <0.0002 |
| SAPS II (median of means, IQR)^†^ | 21.8 (15.2-30.0) | 21.5 (15.0-29.9) | 23.7 (17.6-30.6) | <0.0001 |
| TISS-10 (median of means, IQR) | 9.9 (6.7-12.9) | 10.0 (6.7-13.0) | 8.3 (5.0-11.5) | <0.0001 |
| **Patients treated with antibacterials** | | | | |
| **Characteristic** | **All patients** | **<80 years** | **≥80 years** | **p-value*** |
| Number of patients | 2608 (53.3%^‡^) | 2284 (54.1%^§^) | 324 (48.6%^§^) | 0.0083 |
| Patient days | 35,435 | 32,403 | 3032 |  |
| Length of stay (median, IQR (days)) | 8.7 (4.4-15.8) | 9.1 (4.6-16.8) | 5.8 (3.5-10.8) | <0.0001 |
| Female patients | 976 (37.4%) | 817 (35.8%) | 159 (49.1%) | <0.0001 |
| ICU mortality rate | 620 (23.8%) | 520 (22.8%) | 100 (30.9%) | 0.0014 |
| SAPS II (median of means, IQR)^†^ | 25.3 (18.8-33.6) | 25.2 (18.8-33.8) | 26.2 (19.0-31.8) | 0.8571 |
| TISS-10 (median of means, IQR) | 11.0 (7.7-14.5) | 11.3 (8.3-14.8) | 9.2 (5.0-12.6) | <0.0001 |
| Exposed patient days | 20,659 | 18,789 | 1906 |  |
| Number of Anti­bacterial prescriptions | 128,602 | 115,637 | 12,965 |  |
| Therapy days | 33,679 | 30,670 | 3009 |  |

^*^from Chi^2^-Test for proportions and Mann-Whitney-U-Test for continuous variables (two-sided with significance level of p <0.05)

^†^SAPS II calculated without age points

^‡^Percent of all patients with length of stay > 48 hours

^§^Percent of age group

**Additional file 1: Table S4: R**ate ratios for antibacterial classes (patients with length of stay >48 hours)

| **Drug class** | **Unadjusted rate ratio**  **≥80 vs. <80^‡^** | **Adjusted rate ratio**  **≥80 vs. <80^§^** |
| --- | --- | --- |
| All antibacterials | 1.01 (0.96-1.05) | 1.03 (0.98-1.08) |
| All antibacterials except  erythromycin | 1.07 (1.02-1.13) | 1.09 (1.03-1.14) |
| Tetracyclines | 2.16 (1.50-3.12) | 1.78 (1.16-2.72) |
| Penicillins | 1.44 (1.32-1.57) | 1.40 (1.28-1.53) |
| Cephalosporins | 1.28 (1.16-1.40) | 1.24 (1.13-1.37) |
| Carbapenems | 1.30 (1.12-1.46) | 1.35 (1.21-1.51) |
| Macrolides | 0.98 (0.90-1.06) | 0.98 (0.90-1.06) |
| Clarithromycin | 1.16 (0.58-2.29) | 0.98 (0.43-2.23) |
| Lincosamides | 1.58 (1.16-2.16) | 1.42 (1.00-2.03) |
| Aminoglycosides | 1.17 (0.65-2.11) | 1.14 (0.63-2.07) |
| Fluorquinolones | 1.26 (1.12-1.42) | 1.22 (1.08-1.38) |
| Glycopeptides | 2.23 (1.12-4.44) | 1.49 (0.73-3.03) |
| Imidazoles | 1.41 (1.29-1.55) | 1.40 (1.28-1.54) |
| Oxazolidinones | 1.21 (1.01-1.46) | 1.27 (1.05-1.53) |

^‡^Zero-inflated Poisson-regression

^§^Zero-inflated Poisson-regression adjusted for mean SAPS II- and TISS-scores, sex, and year of treatment
